# Emerging proteomic approaches to identify the underlying pathophysiology of neurodevelopmental and neurodegenerative disorders

**DOI:** 10.1186/s13229-020-00334-5

**Published:** 2020-04-21

**Authors:** Nadeem Murtaza, Jarryll Uy, Karun K. Singh

**Affiliations:** Stem Cell and Cancer Research Institute, Department of Biochemistry and Biomedical Sciences, McMaster University, 1280 Main Street West, Hamilton, ON L8N 3Z5 Canada

## Abstract

Proteomics is the large-scale study of the total protein content and their overall function within a cell through multiple facets of research. Advancements in proteomic methods have moved past the simple quantification of proteins to the identification of post-translational modifications (PTMs) and the ability to probe interactions between these proteins, spatially and temporally. Increased sensitivity and resolution of mass spectrometers and sample preparation protocols have drastically reduced the large amount of cells required and the experimental variability that had previously hindered its use in studying human neurological disorders. Proteomics offers a new perspective to study the altered molecular pathways and networks that are associated with autism spectrum disorders (ASD). The differences between the transcriptome and proteome, combined with the various types of post-translation modifications that regulate protein function and localization, highlight a novel level of research that has not been appropriately investigated. In this review, we will discuss strategies using proteomics to study ASD and other neurological disorders, with a focus on how these approaches can be combined with induced pluripotent stem cell (iPSC) studies. Proteomic analysis of iPSC-derived neurons have already been used to measure changes in the proteome caused by patient mutations, analyze changes in PTMs that resulted in altered biological pathways, and identify potential biomarkers. Further advancements in both proteomic techniques and human iPSC differentiation protocols will continue to push the field towards better understanding ASD disease pathophysiology. Proteomics using iPSC-derived neurons from individuals with ASD offers a window for observing the altered proteome, which is necessary in the future development of therapeutics against specific targets.

## Autism spectrum disorders

Autism spectrum disorders (ASD) are a broad range of neurodevelopmental disorders, ranging in severity between individuals. It is defined by two core symptoms, impaired social communication and reciprocal interaction, and the presence of repetitive behaviors and restricted interests. Due to heterogeneity in ASD, treatments are currently focus on the associated symptoms of ASD, specifically irritability and aggression through either risperidone or aripiprazole, both originally prescribed as antipsychotics [1]. However, elucidation of biological pathways underlying ASD is required before new therapies can be developed. In this review, we explore emerging technical advances in proteomics that provide new tools to gain insight into novel and clinically relevant ASD signaling networks, which can be applied to models, such as patient-specific induced pluripotent stem cells (iPSCs).

## Current research approaches in ASD

**Fig. 1:**
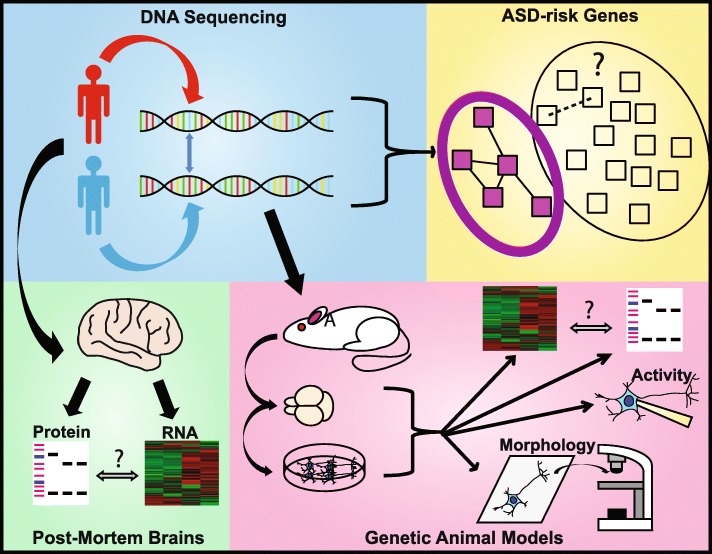
**Current research in ASD**. Current ASD research focuses on genetic sequencing studies to identify ASD risk genes, based on the enrichment of single nucleotide or copy number variations. Following identification, post-mortem brains from individuals with ASD are used for analysis of the proteome or transcriptome, and single genes are studied using animal models. Animal models are studied in vivo and in vitro for changes in neuron morphology and activity as well as in the transcriptome and proteome. Post-mortem brains are limited in availability and do not provide insight into the early developmental time points directly, while animal models only study one gene at a time, resulting in the low throughput elucidation of disease-relevant mechanisms for only a minority of ASD-risk genes

Large-scale genome sequencing studies have identified numerous ASD risk genes (reviewed by Iakoucheva et al. [2]), which have for the most part, been studied using animal and human models [3] (Fig. 1). Many major cellular pathways have been associated with ASD pathophysiology, including growth and activity, synaptic transmission, excitatory/inhibitory balance, plasticity, protein synthesis, and neuron-glia signaling (reviewed by Chen et al. [3]), and metabolic signaling and mitochondrial function (reviewed in [4]; however, studying individual genes/pathways is a time-consuming and a costly process. In addition, human imaging or post-mortem studies have pinpointed neuroanatomical and brain connectivity differences. For example, structural magnetic resonance imaging (MRI) and diffusion tensor imaging (DTI) revealed decreased connectivity in the corpus callosum, structural shifts and increased activity in the frontal lobe, and altered connectivity across cortical regions and within the limbic system involved in memory and emotions (reviewed in [3, 5]). A recent study showed an increase in the metabolic rates of neurons crossing the corpus callosum in individuals with ASD and SCZ [6]. Post-mortem imaging studies have identified phenotypes including, atypical cortical column development, altered neuronal density in cortical layers II/III and V, and increased inflammation and glial activity (reviewed in [3, 5]). These studies reveal aberrant connectivity between regions of the brain, especially in the cortex and cerebellum, which are highly associated with ASD.

**Fig. 2:**
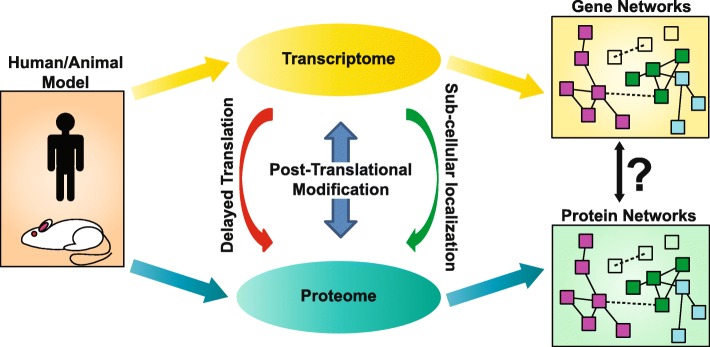
**Identification of molecular networks using the transcriptome and proteome**. Investigation into the transcriptome and proteome in both human and animal models have resulted in biological networks based on RNA and protein. Differences between the transcriptome and proteome due to delayed translation, post-translational modificaton, and sub-cellular localization could lead to discordance between these two sets of networks

Studies of the transcriptome have been key for identifying disrupted networks [7, 8] (reviewed by Quesnel-Vallieres et al. [9]), but a caveat of these studies is the discordance between mRNA levels and protein levels [10]. Although steady-state mRNA does correlate with protein, and both can be used to distinguish cell and tissue types [11, 12], it does not directly match protein expression levels [13, 14]. Perturbations to the cell can drastically shift the correlation between mRNA and protein due to delayed mRNA translation, differential sub-cellular localization of mRNA, and post-translational modifications (Fig. 2). Very few studies in the ASD field have analyzed the proteome and therefore the actual changes in protein levels are not fully understood. Furthermore, post-translation modifications (PTMs) can greatly affect protein activation, binding affinity, and folding and turnover rates [15, 16] (Fig. 2). Transcriptome research remains an important part of ASD investigations; however, the proteome is still greatly unmapped and has the potential to drastically advance our understanding of neurological disorders.

## Altered biological processes in autism spectrum disorders

ASD-associated genes can also be categorized into many major gene networks including cytoskeleton, channels, post-synaptic density, chromatin, and intra-cellular signaling [17, 18]. A recent study shows that ASD-associated genes can be grouped into two broad and distinct categories, gene regulation and neuronal communication [19]. Multiple biological processes have been associated with ASD through studying genetic animal models [3]. Most studies use large annotated databases, such as Gene Ontology, KEGG, or REACTOME, to identify the biological processes enriched by ASD risk genes. These networks are generally based on meta-databases that rely on known pathways and interactions. However, they rely majorly on RNA co-expression datasets that do not reflect the changes at the protein level, or on interactions identified in non-neuronal or non-mammalian models. This is further the case with RNA sequencing and single-cell RNA sequencing, where many pathways are identified through the transcriptome. Mass spectrometry is a versatile technique that allows the study of the entire proteome and to date has only been used minimally to study ASD. Thus, we will focus on different approaches that use mass spectrometry to study changes in the proteome.

## Proteomic approaches to study signaling networks

Mass spectrometry (MS) is the popular choice to study proteomics due to its ability to handle complex protein samples with high resolution. It is highly versatile and can be applied to study protein abundance, PTMs, and protein interactions. Proteins from biological samples are ionized into fragments and then into precursor ions for detection by a specific mass analyzer. The two most common ionization tools are liquid chromatography-electron spray ionization (LC-ESI) and matrix-assisted laser desorption ionization (MALDI) [20]. LC-MS is typically the preferred method for analyzing complex protein/peptide mixtures; on the other hand, MALDI-MS is used to analyze simple peptide mixtures (< 100 proteins), such as blood, urine, and saliva [21].

## Quantitative proteomics

**Fig. 3:**
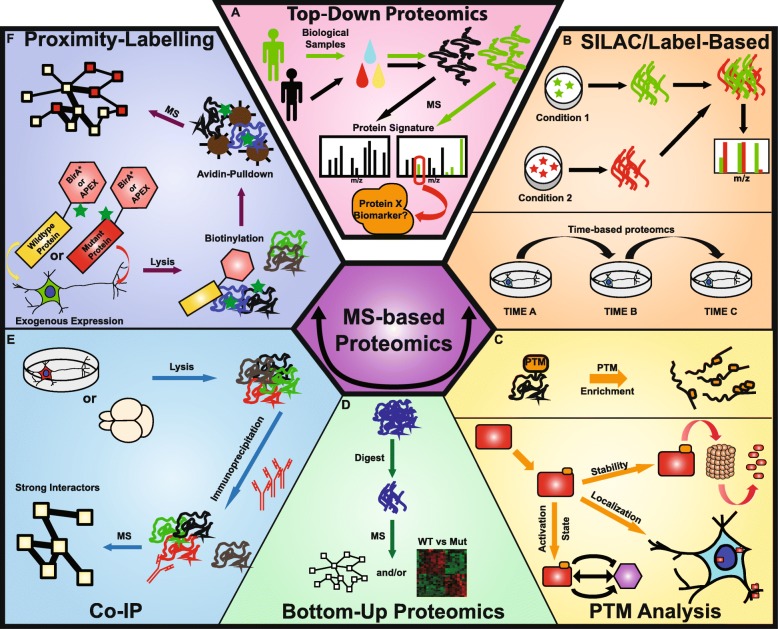
**Studies in neurodevelopmental disorders using MS-based proteomics**. (**a**) Top-down proteomics allows the identification of protein signatures and specific proteins altered in biological samples, such as blood, urine, and saliva, from affected and unaffected individuals. (**b**) SILAC and label-based proteomics can be used to combine multiple samples to reduce variability and allow normalization across samples, presenting a powerful tool for time-course based studies in vitro and in vivo. (**c**) PTM analysis allows the identification of protein modifications that regulate the activity, localization, and stability of the protein. (**d**) Bottom-up proteomics relies on the digestion of proteins into peptides to allow more precise and accurate quantification and identification through mass spectrometry. It allows for the use of SILAC and label-based proteomics, identification of PTMs, and the ability to identify PINs using proximity-labeling or Co-IP. (**e**) Co-IP uses antibodies or affinity tags to isolate endogenous proteins and identify strongly interacting proteins that are simultaneously pulled-down, through mass spectrometry. (**f**) Proximity labeling uses the expression of a protein of interest fused to a labeling-protein, such as BirA* or APEX, which biotinylates proteins in close proximity. These proteins, which include strong and transient interactors, can then be isolated using an avidin pull-down and identified through mass spectrometry. Proximity labeling can allow for the comparison between wildtype, mutant, or condition-treated PINs

There are two methods of quantitative proteomics, top-down and bottom-up (Fig. 3a, d). Top-down proteomics is generally used to analyze simpler protein mixtures and allows for high coverage and characterization of a protein’s “proteoform,” the variable form of a protein due to genetic variation, alternate splicing, and PTMs [22]. Top-down proteomics is an excellent choice when investigating specific proteins of biological importance to observe changes in the proteoform, possibly by PTMs. However, it lacks proteome-wide coverage, sensitivity, and high-throughput capacity limiting its usage to pure or simple protein samples. The majority of workflows are bottom-up, also called shotgun proteomics [23]. Tandem-MS (MS/MS) is typically used in bottom-up proteomics, and LC-MS/MS is the most common method for quantifying proteins on a global scale. The high sensitivity allows for the detection and quantification of low abundant molecules, specific PTMs, and protein-protein interactions, and the characterization of subcellular compartments [24]. In contrast to top-down methods, bottom-up strategies can have higher coverage and increased high throughput and multiplexing of samples.

## Label-free protein quantification

Label-free quantification (LFQ) is a MS-based proteomic method that determines the relative or absolute amount of proteins between biological samples. LFQ methods are able to achieve higher sequence coverage than label-based methods, allowing for highly reliable protein identification [25]. Due to the ability to run multiple samples without limit, it is excellent for identifying changes in multiple cell and tissue types. One study tracked changes in the proteome of old and young mice with Alzheimer’s disease (AD) pathology and found that there was an increase of amyloid beta proteins coupled with a decrease in AMPA receptor trafficking proteins over time [26]. It has also been used for bottom-up proteomics, including the region-specific analysis of the *Shank3* mouse model of syndromic ASD [27]. However, LFQ is more prone to instrumentation error and variability between runs, which can be reduced through standard or sample-spiking strategies [28].

## Label-based protein quantification

Label-based proteomics is achieved using chemical or metabolic tags. The most popular choice are the isobaric tags for relative and absolute quantification (iTRAQ) [29, 30]. These tags can be used to multiplex up to eight samples. Similar to iTRAQ is the tandem mass tag (TMT), which can multiplex up to 16 different samples [31]. Chemical labeling allows for samples to be mixed together as “one” sample and run at the same time, which allows for comparison between samples and reduces variability (Fig. 3b). However, there are some practical drawbacks including the labeling efficiency, which may fluctuate between samples. Technical differences introduced by isobaric labeling can also be avoided by using the alternative metabolic labeling.

## Metabolic labeling of proteins through stable isotope labeling with amino acids (SILAC)

The most conventional metabolic labeling technique is stable isotope labeling with amino acids in cell culture (SILAC), which involves feeding cells media containing isotopic amino acids [32] and can be mixed for subsequent processing for mass spectrometry (Fig. 3b). This allows for detection of differences in protein abundances in cell cultures and can be modified to examine de novo protein synthesis [33, 34]. SILAC has been used to study neurodevelopmental and neurodegenerative disorders [35, 36]. SILAC on primary cultures of *Fmr1* KO mice cortical neurons showed decreases in synaptic proteins and mRNA transport [37]. Tang et al. used the same technique and showed that loss of Fmr1 may have a larger impact during early postnatal brain development, showing an increase of pre- and post-synaptic proteins [38]. Furthermore, SILAC has been used to study ASD-associated de novo mutations in the E3 ubiquitin ligase, UBE3A. Profiling of wild-type UBE3A or the T485A mutant using SILAC-MS combined with ubiquitin affinity profiling determined that mutant UBE3A interacts with and ubiquitinates multiple subunits of the proteasome complex, decreasing its stability and causing overactive Wnt signaling [35]. While SILAC has not been employed widely in other ASD-associated models, using it on genetic knock-out or knock-in models can identify changes in the neuronal proteome over time (Fig. 3b). While most studies are still done in vitro, progress has been made for in vivo SILAC labeling [39–43].

## Identifying and quantifying post-translation modifications

PTMs are important as they can alter a protein’s spatial-temporal function by changing its conformation, activity, or stability (Fig. 3c). Proteomics, unlike genomics, allows the opportunity to directly identify PTMs in a selective manner through specific purification methods [44]. Alterations in PTMs have been implicated in multiple neurodegenerative disorders [45, 46]. There are many types of PTMs, including phosphorylation, methylation, palmitoylation, glycosylation, acetylation, SUMOylation, ubiquitination, and S-nitrosylation. Phospho-proteomics can map differential phosphorylation of kinases and phosphatases and their substrates, and it has been used to identify novel altered pathways in AD, ASD, and other neurological disorders [47]. For example, investigation of the phospho-proteome in AD cell line models and post-mortem brain samples has revealed the involvement of specific kinases in heat shock protein-dependent protein folding, insulin-mediated signaling, p53 regulation, and neuronal autophagy [48–50]. Studying the relevant ASD phospho-proteome, SILAC-MS of mouse *Fmr1* KO mouse embryonic fibroblasts revealed multiple changes to the MAPK, mTOR, Wnt, and p53 signaling pathways [51]. Although these pathways were previously linked to altered translation and neurogenesis in ASD, they showed that differential expression and phosphorylation of multiple proteins are involved in each pathway. In addition to global changes, phospho-proteomics can detect changes in specific cellular compartments. Collins et al. published one of the first mouse synapse phospho-proteomes and identified 331 phosphorylation sites in the pre- and post-synapse [52]. More recent work identified 1554 phosphorylation sites in the post-synaptic density, which are altered during neuronal stimulation [53]. These studies highlight the complex regulation of synaptic proteins that is missed using transcriptomics. In addition to phosphorylation, an investigation using *Shank3* mutant mice found large changes in the S-nitroso-proteome, which altered vesicle release at the presynaptic terminal [54]. Interestingly, changes in the S-nitroso-proteome has also been associated with a tauopathy model of AD [55]. Methylation of histone complexes is another topic of interest, as many ASD genes are linked to chromatin remodeling. For example, SETD5 happloinsufficiency revealed that SETD5 methylates histones directly, suggesting an important role of H3K63 methylation, which in turn regulates RNA elongation and processing [56]. Together, these studies emphasize how important PTMs are for protein regulation and function.

## Identifying protein-protein interaction networks (PINs)

Proteomics studies can provide direct information about the altered protein levels in genetic mouse models of ASD; however, they cannot identify the networks that are directly regulated by the protein. The increasing number of genes identified in neurodevelopmental disorders highlight the obstacles in identifying relevant pathways [35, 36]. Traditionally, protein-protein interactions (PPIs) were identified using two-hybrid screens; however, there are now multiple proteomic-based methods, such as affinity pull-down or co-immunoprecipitation and proximity labeling, which are discussed here.

Two-hybrid (2H) screening is used as a confirmation tool to determine if two proteins are in close proximity or interacting directly [57]. This method uses both bait and prey proteins that are fused to separate fragments, which when in close enough proximity come together to produce an output. In 2011, Sakai et al. used yeast two-hybrid (Y2H) with a human cDNA library of 192 bait fragments and identified 848 interactions with 26 syndromic ASD or ASD-associated proteins, where only 32 interactions were previously known [58]. Their screen identified proteins localized to the synapse, post-synaptic density and the cytoskeleton, that involved small GTPase-mediated signaling and metabotropic glutamate receptor signaling, demonstrating the utility of Y2H. Corominas et al. later used Y2H on 422 splicing isoforms from 168 autism candidate genes and identified 506 protein-protein interactions between 71 bait and 291 prey proteins [59]. They found that one third of the gene-level interactions would not be identified if only using the reference isoform, suggesting a strong role for altered splicing in ASD. Although 2H screens provide direct evidence of interactions, they are only identified between fragments used in the screen, therefore biasing results. 2H methods also generally require the use of less complex organisms and it is not adapted for human cell models; however, new proteomics technologies may be better suited for detecting novel PPIs in human cells.

### Affinity pull-down and co-immunoprecipitation

Affinity pull-down or co-immunoprecipitation (Co-IP) coupled with mass spectrometry is an approach to identify interacting proteins (Fig. 3e). After cell lysis and pull-down using an antibody, mass spectrometry can be used for protein identification. In some cases, the protein of interest can be fused to an affinity tag [60]. IP-mass spectrometry has been used to identify the interacting proteins for important neuronal channels including AMPA and kainate receptors [61]. The Grant group used this method to identify the interactome of the NMDA and AMPA receptors and the PSD95 scaffold protein [62–64]. Additional investigation of the PSD using biopsies and post-mortem brain samples revealed that 14% of proteins in this compartment are associated with nervous system disorders and were highly conserved between species [65]. Bayes et al. further described an increased association between MAGUK-associated proteins and ASD within the synaptic compartment [66], while a study of the mouse PSD proteome during two developmental time points revealed differential association between PSD95 and SHANK3 [67]. These studies were the first to describe a strong connection between an altered synaptic proteome and ASD. Using Co-IP, Berg et al. identified the PIN of JAKMIP1 in mouse cortical neurons, a gene that is differentially expressed in patients with Fragile X syndrome and dup(15q11-13) syndrome [68], demonstrating that it is involved in translation, including FMRP and FMRP targets. Co-IP also identified new functions of proteins within specific cellular compartments. Cytosolic DYRK1A was originally associated with cell cycle and cytoskeleton organization [69, 70], but a novel function for DNA damage repair was shown in the nuclear compartment [71]. In addition, the functional impact of genetic mutations was described showing ADHD-linked mutations in the Na+/H+ exchanger 9 protein (encoded by *SLC9A9*) alters the interaction with caveolae-mediated endocytosis and MAP2K2-mediated signaling [72]. Finally, 11 ASD-associated genes were investigated using IP-mass spectrometry and shared signaling mechanisms between FMRP and MECP2, and idiopathic and non-idiopathic ASDs were discovered [73]. Together, these studies highlight the value of using Co-IP mass spectrometry as an important tool in identifying PINs.

Technical limitations of Co-IP include the strength of the interaction after cell lysis, quality of the antibody, and the use of appropriate controls. However, techniques such as chemical cross-linking mass spectrometry have been used to identify more transient interactors in the synaptic compartment [74]. Co-IP works well with scaffold and adherent proteins that are enriched in the synaptic compartment (e.g., SHANK or HOMER). Although cross-linking is a potential method of trapping transient interactions, it can also introduce artifacts. A Co-IP study of 16 proteins in seven ASD mouse models was able to cluster the models based on synaptic interaction strength and successfully predicted deficits in the AKT pathway in the *Ube3a* mouse model [75]. Although this study was not coupled to mass spectrometry, it suggests the potential of the method for concurrently studying different models of ASD using Co-IP/MS.

### Proximity-based labeling of proteins

Proximity-based protein labeling coupled to MS has been a ground-breaking method for identifying protein-protein interactions (PPIs) [76]. It allows the screening of endogenous PPI networks in live cells (Fig. 3f). Labeling proteins in cells prior to harvesting reduces artifacts due to presence of detergents and isolation steps. Proximity labeling has the advantage of identifying weak and transient interactions, which are common in metabolic and intracellular signaling. The method requires the proximity labeling of neighboring proteins, rather than direct physical protein interactions, which can be identified long after the interaction has ended.

There are two major methods, engineered ascorbate peroxidase (APEX) or the promiscuous BirA* biotin ligase (BioID) [77, 78]. BirA* uses ATP to create active biotin that labels lysine residues, while APEX utilizes biotin-phenol that is activated by hydrogen peroxide and labels tyrosine [79]. Biotinylation allows the labeling of nearby proteins that can be pulled down using affinity chromatography against biotin and run through the mass spectrometer. APEX and BioID have mainly been used to identify the proteins in cellular compartments or signaling pathways in mammalian cells lines such as HEK293 cells [80–86]. Only two studies have used APEX on cultured cortical neurons, focusing on the identification of proteins in the synaptic cleft or the identification of the RNA localized to different cellular compartments [87, 88]. These studies required a large number of neurons (> 90 million cells), possibly due to quick activation dynamics. The development of proximity-labeling methods to reduce the number of primary cells required would increase throughput of testing different NDD risk genes to build comprehensive PPI networks. A recent BioID study used hippocampal cultures and mapped the axon initiation segment (AIS) by BirA* tagging multiple AIS proteins, including NF186, Ndel1, and Trim46. They identified previously unknown proteins necessary for proper AIS formation, such as Mical3, although some known AIS associated proteins were not identified [89]. In the first use of BioID in vivo, PSD95 (excitatory synapses) or gephyrin (inhibitory synapses) was fused to BirA* and expressed in the mouse brain, identifying known and novel interactors [90] and revealing new inhibitory signaling. Another study from the same group later characterized the interactome of CARMIL3 and showed it interacted with cytoskeleton proteins in the synapse as a new synapse regulator [91]. However, in vivo BioID requires the use of adeno-associated virus that has smaller packaging size limitations, preventing the study of larger proteins, many of which are encoded by ASD risk genes and expressed in the brain.

Although proximity labeling is a powerful approach, users should be aware of limitations. APEX requires the addition of hydrogen peroxide and cannot be used in vivo due to toxicity. Tyrosine residues are among the least abundant amino acids in and therefore could result in missed labeling [92]. Alternatively, BioID only requires ATP and biotin, which are found in all cells and labels one of the most common amino acids, lysine. BioID offers more versatility using in vitro or in vivo models and has a higher chance of labeling all proteins within its active radius. However, the incubation and label time required is 15–24 h, while APEX requires only 30–60 min and can label proteins within a minute [93]. For these reasons, BioID is generally used to identify a history of interacting proteins, while APEX can capture interacting proteins during a short period of time. A newer version of BioID, called TurboID, allows biotin labeling within a 10-min time frame, creating the opportunity to study dynamic changes in PINs [94].

BioID has not yet been reported to be used in iPSC-derived neurons or brain organoids where it will be very useful to identify distinct and shared human signaling networks. It is important to remember that endogenously biotinylated proteins are always present and could create a background of biotinylated proteins. The proper controls are therefore required to compare the experimental condition to a control [87]. However, overexpression of the protein of interest can alter the state of the cell, and therefore, a proper control should express the protein of interest, as well. The use of self-cleavage 2A sequences may be beneficial because it allows the expression of the protein and proximity-labeling enzyme simultaneously and separately, so that both conditions only differ in the protein fusion [95].

## Proteomic analysis of post-mortem brains from individuals with ASD

Studying neurological disease in the human brain is difficult due to the inability to acquire live samples. Post-mortem brains offer a way to study the human brain directly and have been used in a limited fashion to study ASD [96–98] (Fig. 1). RNA sequencing analysis of post-mortem ASD brain samples revealed multiple significant changes in long non-coding RNAs, gene splicing events and gene expression [7, 99]. However, the correlation between the gene expression and protein expression is not fully understood. In one of the first uses of proteomics on post-mortem ASD brain samples, two brain regions, Brodmann area 19 (BA19) and the cerebellum, were investigated in idiopathic ASD. They identified distinct proteomes of each region and pathway analysis revealed changes in synaptic scaffold, glutamatergic transmission, calcium signaling, and neurofilament proteins; however, the proteomic signatures of both regions were not different between controls and ASD brains. This data refutes previous RNA sequencing data from postmortem studies and suggests there are brain regional molecular differences in autism. Moreover, the proteomic data in this study unexpectantly pointed to potential dysregulation of protein expression in ASD brains might be through upstream regulates that have common signailng pathways with neurodegeneration. Another study by Broek et al. used mass spectrometry to measure 14 chosen proteins in the prefrontal cortex (PFC) and the cerebellum of ASD and control individuals [96]. They also identified opposing regional differences in proteins related to myelination, synaptic function, and energy metabolism, but a similar decrease in the astrocyte marker protein, vimentin [100]. It is worth nothing that multple post-mortem proteomic studies have been undertaken for schizophrenia and bipolar disorders, revealing similar dysregulated pathways [101–103]. Limitations to using postmortem tissue include the limited availability of subjects or age-matched controls, exposure to alcohol or other neurological treatments that may alter the proteome, and post-mortem tissues have already begun the process of cell death that can confound results. In this regard, Bayes et al. describe differences in protein integrity between live biopsy and post-mortem samples between different regions in the brain and found that protein complexes in the synaptic compartments are more stable than other compartments [66]. To counteract these limitations, immotalized cell lines, such as subject lymphoblastoid cell lines, can be used. Researchers identified 82 proteins altered in ASD subgroups that have sever language impairment and reductions in the diazepam-binding inhibitor protein correlated with the autism diagnostic interview-revised scores [104]. However, these types of cell lines are difficult to interpret because they are non-neuronal.

## Proteomic studies to identify biomarkers using live patient biological specimens

The limitations in obtaining the human brain historically has forced studies to use easily accessible samples, including urine, blood, and saliva. Analysis of urine identified changes in metabolites, toxins, and proteins as potential markers associated with ASD, but the relevance remains in question because the samples contain salts, which may skew mass spectrometry results. Blood offers a live biological samples for mass spectrometry, where MALDI-TOF was used to identify proteins that are changed in ASD (or subtypes of ASD) in several studies [105–107]. More specifically, differences in protein signature between subgroups of Rett syndrome have been identified [107]. Bottom-up proteomics have revealed consistent changes in proteins involved in mitochondrial function, ER stress and protein folding, endocytosis and immune response, and metabolites, including in children with mental regression [108, 109]. Wei et al. identified reduced levels of the STOP/MAP6 protein in the blood plasma of autistic children [110]. Similar analyses of urine and saliva have identified metabolites and compounds altered in individuals with ASD [111–114]. Proteomic analysis of SCZ and psychosis patient samples have also identified possible proteomic signatures, highlighting its use to identify disease biomarkers for both NDDs and other neurological disorders [115, 116]. The major disadvantage to using these biological samples is the innate differences of cell types found within the blood, lymphatic, and urinary systems, which can potentially introduce proteins not relevant to the disease.

## Using human induced pluripotent stem cells (iPSCs) to study ASD

Human iPSCs allow the study of human neurological disorders with a human genetic and biological background, which is not possible in animal models [117, 118]. Human iPSCs are routinely now generated using skin fibroblasts or blood cells [119–122]. There are some considerations when using iPSCs, for proteomics studies. The differentiation of iPSCs into neurons may produce mixed cell types, which can confound proteomic findings [117, 123–125]. Furthermore, human iPSC-derived cells cannot fully address the altered brain connectivity observed in neurodevelopmental disorders, although co-culturing of multiple human-derived cell types or 3D organoids can provide more complex systems. In addition, large numbers of iPSCs are needed to generate sufficient neural cells for proteomic studies, which can be cost prohibitive. However, with recent advances in mass spectrometers, the amount of sample needed for signal has been steadily declining [126, 127].

### Proteomic analysis of 2D human iPSC-derived neuron cultures

Targeted mass spectrometry of neurons differentiated from iPSCs identified increased expression of pre- and post-synaptic proteins (e.g., STXBP1, SYN1, VAMP2, GRIA1, and SYNGAP), suggesting that major neuron-specific proteins can be identified [128]. In an investigation of mutations in MECP2 associated with Rett syndrome, SILAC-MS was used to reveal that NPC-derived neurons with the Q83X or N126I mutations exhibit a downregulation of multiple astrocytic markers (ALDOC, S100B GFAP) in 3-week-old neural cultures [129]. Further investigation using SILAC revealed that NPCs lacking MECP2 have increased expression of LIN28, which is known to repress differentiation into glial cells [130]. A recent study using iPSC neuron of Rett syndrome patients identified 4 subsets of proteins that were differentially expressed, across time points involved in filipodia assembly, synapses, axon guidance, and cytoskeleton and translation [131]. This showed distinct temporal deficits during neuronal development, highlighting the fluid pathology underlying Rett syndrome.

Mass spectrometry to identify PTMs of proteins has also been applied to neurodegenerative models. PTMs in the tau protein were found using iPSCs generated from individuals diagnosed with frontomporal dementia and possessing the A152T mutation in *MAPT* (which encodes for the tau protein) [132]. They characterized differences in MAPT splicing and tau PTMs, showing that A152T neurons had increasing levels of tau. Another group identified altered phosphorylation and cysteine modifications in the cytoskeleton and RhoA signaling proteins in dopaminergic neurons with loss of PARK2 [133]. Further, when comparing differentially expressed proteins in spinal muscular atrophy iPSC-derived motor neurons to their source fibroblasts, the iPSC-derived motor neurons had fewer proteins than controls, including proteins involved in viability (beta III-tubulin and UCHL1) and UBA1 involved in protein degradation signaling [134]. This study showed an important role for SMN (mutated in SMA) in the differentiation capacity of iPSCs to produce motor neurons.

The same methodology can be used to track the changes in the proteomic profile of other differentiation protocols. Varderidou et al. used two iPSC to neuron differentiation protocols, expression of NGN2 to generate excitatory neurons and small molecules to generate motor neurons [135]. Interestingly, they compared both the proteome and transcriptome during neuronal differentiation and found that both methods have different signatures, highlighting significant differences between the RNA and protein levels within human engineered neurons.

Mass spectrometry has also been used to study the proteome of non-neuronal brain cells. Differentiation of iPSCs from patients with Costello syndrome, a neurodevelopmental disorder, casued by heterozygous mutations in RAS (HRASG12S), revealed an increased differentiation and maturation into astrocytes. To identify the extracellular proteins produced by mutant astrocytes, shotgun mass spectrometry was used to identify an enrichment of extracellular matrix remodeling proteins and proteoglycans, which have an important role during critical periods of maturation and synaptic plasticity [136]. Finally, quantification of proteins in the cultured media of iPSC-derived neurons provides a potential avenue to identify disease biomarkers. For instance, reduced levels of ORM1 have been found in the culture media of iPSC-derived neurons from patients with AD, which coincides with reduced ORM1 levels that have been found in the cerebrospinal fluid of AD patients [137]. This finding highlights the utility of identifying disease biomarkers through mass spectrometry of iPSC-derived neuron media [137].

### Proteomic analysis of 3D human iPSC-derived neuron cultures

Advancements in neural differentiation of iPSCs has allowed for the generation of 3D human neuron cultures, known as organoids [138–140], which have better maturation profiles. Multiple different brain region-specific organoids can be generated with a combination of sequentially introduced chemical modulators [141], allowing better recapitulation of brain development, making them a critical system for evaluating neurodevelopment [142]. Nascimento et al. used label-free shotgun proteomics to study human cerebral organoids and identified > 3000 proteins from neuronal progenitors, neurons and glial cells, and proteins relevant to neurodevelopment including axon guidance, synaptogenesis, and cerebral organoids [143]. Furthermore, disease-specific brain organoids were derived from patients with AD, subjected to LC-MS/MS, identifying altered proteins belonging to axon growth, mitochondrial function, and antioxidant defense [144]. Analysis of post-mortem brain tissues from AD patients showed similar findings indicating the power of the organoid culture system using proteomics. Another study examined how the 16p13.11 microduplication affected neurodevelopment using cerebral organoids [145]. Transcriptomic analysis of patient-derived cerebral organoids showed perturbations in the NFkB p65 pathway, confirmed through proteomic analysis and highlights the growing interest to combine proteomics with patient iPSC-derived cerebral organoids in studying NDDs. We predict this system will be used further given the advanced maturation in patient-derived organoids, where proteomic-based methods can be used to identify the complex disease signaling networks.

## Statistical analysis of proteomic data

Mass spectrometry requires the mapping of peptides and proteins, based on their spectral signature, to annotated protein databases (reviewed in [146, 147]). However, the matching of spectra between an experimental dataset and the protein/peptide database allows the possibility of incorrect identifications. Proteins identified have an associated minimal false discovery rate for incorrect identification, allowing for lenient or strict identification. Furthermore, due to the similarities between multiple proteins, especially isoforms and proteins of the same family, most studies use a minimal cutoff of 2 unique peptides for the identified proteins. There are many analysis tools for proteomic data, including Mascot, SEQUEST, Patternlab, MaxQuant, and Saint analysis; however, there is no standard for analysis, which may contribute to the variability between studies [148–152]. These proteins will then be questioned in regard to protein expression levels, modification, enrichment of networks and pathways, or changes in protein interaction networks. There are multiple methods of determining differences in protein levels, such as identifying outliers based on a fitted curve or standard deviation of a peptide ratio vs. abundance curve [150, 153]. Schmidt et al. offer a brief review of multiple software packages that can be utilized for analyzing mass spectrometry data [154].

## Overall challenges and limitations of proteomics

Variability remains a major challenge in proteomic studies of neurological disorders. In fact, a meta-analysis of 87 synaptic proteomic studies found that only 6% of all dysregulated proteins were the same across studies. However, when looking at proteins similarly affected across experiments, proteomic signatures could be identified for multiple neurological disorders [155]. Another major challenge is the heterogeneity of neurological disorders, which necessitates the use of strict criteria when choosing subjects. Major differences in these aspects can cause large variations in the proteome. Another technical challenge is the reduced sensitivity to detect low abundance proteins and splice isoforms. It also requires the use of uniform cells to identify robust changes in the proteome and therefore relies on good cell isolation techniques. Further, sample preparation presents a major source of variability, including loss of protein during processing and differences in trypsinization or desalting [156]. This issue can be overcome in some cases using SILAC-based MS or the use of isobaric labeling. Labeling across all biological samples and replicates avoids the inherent variability of the MS instrumentation. The labeling of peptides also allows for combining all samples to be run as one, which reduces run time and variability; however, this presents a new problem, in which isobaric tags still have batch-to-batch variation [157]. A recent TMT-labeling study with human iPSCs looked at multi-batch effects and found that reproducibility of a single multiplexed TMT batch drastically falls when integrated with multiple TMT batches and a reference line is incorporated for normalization across runs [158]. Although many factors both methodological and experimental can create variability in mass spectrometry data, one tool is selected reaction monitoring mass spectrometry to validate specific proteins, for example, in post-mortem brains of ASD and control individuals [96]. Here, the majority of peptides and proteins were validated but the presence of some proteins did not match shotgun proteomics, revealing the potential for false negative and positive results.

## Conclusions

**Fig. 4:**
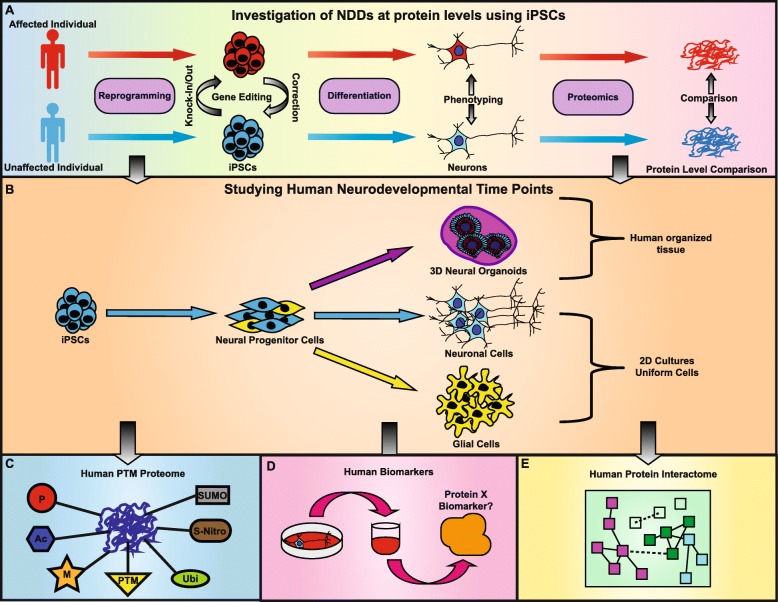
**Investigation of NDDs using human iPSCs and proteomics**. (**a**) Biological samples from affected and unaffected individuals can be reprogrammed into iPSCs, which can be genetically edited to correct potential disease-relevant mutations or introduce gene disruptions and/or patient mutations. These iPSCs can then be differentiated into multiple cell types including neurons and then compared at the cellular and proteome levels. (**b**) The use of human iPSCs allows for the investigation of the proteome at different time points during development, including the iPSC stage, the neural progenitor stage, and the fully differentiated stage. Neuronal and glial 2D cell cultures can be studied, as well as 3D neural organoids that mimic human brain development. Proteomics with iPSC-derived neurons provides the potential to identify human PTM programs (**c**), human disease biomarkers (**d**), and human PINs (**e**)

The majority of iPSC studies on ASD are focused on genomic sequencing; however, there is much less research done at the protein level. The coupling of fractionation with mass spectrometry has drastically increased the resolution between proteins and the accuracy of quantification; however, future improvements to mass spectrometer technology will allow for reduced amounts of required sample. In this review, we provided multiple examples of how proteomics can be applied to study neurological disorders with iPSCs, as shown in Fig. 4. Using iPSCs, studying the proteome at different developmental time points in human neurons or glial cells is now a possibility (Fig. 4b). The ability to identify intracellular and extracellular protein levels and PTMs will drastically advance our knowledge of any brain disorder, including ASD (Fig. 4c, d). Further, 3D human brain organoids and spheroids improve the ability to study proteomic changes of an organized tissue that better mimics the human brain (Fig. 4b). However, the field still lacks the ability to perform single-cell proteomics with high precision and resolution. With proximity labeling, there will be a major focus on determine the protein interaction networks (PINs) for various neurological diseases (Fig. 4e), since this is still a poorly investigated area. Future studies should combine human iPSC-derived neurons with proximity labeling to identify human-specific PINs, which have been lacking due to the difficulties associated with human postmortem brain tissue. In conclusion, mass spectrometry opens major avenues of research into the proteome and the use of human iPSC-derived neural cells presents the potential to study the human proteome. By combing these tools, we can attain far-reaching advancements in understanding the pathophysiology of neurodevelopmental and neurodegenerative neurological disorders.

## Data Availability

Not applicable

## Abbreviations

ASD: Autism spectrum disorder
iPSC: Induced pluripotent stem cells
NDD: Neurodevelopmental disorder
SCZ: Schizophrenia
PTM: Post-translation modification
MS: Mass spectrometry
LC-ESI: Liquid chromatography-electrospray ionization
MALDI: Matrix-assisted laser desorption/ionization
MS/MS: Tandem mass spectrometry
LFQ: Label-free quantification
AD: Alzheimer’s disease
iTRAQ: Isobaric tags for relative and absolute quantitation
TMT: Tandem mass tag
SILAC: Stable isotope labeling by/with amino acids in cell cultures
PPI: Protein-protein interaction
2H: Two-hybrid
Y2H: Yeast two-hybrid
Co-IP: Coimmunoprecipitation
APEX: Ascorbate peroxidase
BioID: BirA* biotin ligase
NPC: Neural progenitor cells
PIN: Protein interaction network
SMA: Spinal muscular atrophy
